# Cattle owners' awareness of bovine tuberculosis in high and low prevalence settings of the wildlife-livestock interface areas in Zambia

**DOI:** 10.1186/1746-6148-6-21

**Published:** 2010-04-20

**Authors:** Musso Munyeme, John B Muma, Hetron M Munang'andu, Clovice Kankya, Eystein Skjerve, Morten Tryland

**Affiliations:** 1 Department of Disease Control, The University of Zambia, School of Veterinary Medicine, PO Box 32379 Lusaka, Zambia; 2 Department of Basic Sciences and Aquatic Medicine, Section of Aquatic Medicine and Nutrition, Norwegian School of Veterinary Science, PO Box 8146 Dep, 0033 Oslo, Norway; 3 Department of Veterinary Public Health, Faculty of Veterinary Medicine Makerere University, PO Box 7062 Kampala, Uganda; 4 Department of Food Safety and Infection Biology, Norwegian School of Veterinary Science, PO Box 8146 Dep, 0033 Oslo, Norway; 5 Department of Food Safety and Infection Biology, Section of Arctic Veterinary Medicine, Norwegian School of Veterinary Science, Stakkevollveien 23, N-9010 Tromsø, Norway

## Abstract

**Background:**

Awareness of bovine tuberculosis (BTB) by cattle owners is of extreme importance to policy makers when considering mitigation. However, to our knowledge, little is known on cattle owners' awareness of BTB in Zambia. Similarly, such knowledge is uncommon within and outside Africa. The current study investigates the epidemiological characteristics of BTB in Zambian cattle in relation to awareness by cattle owners in high and low cattle BTB prevalence settings. A cross sectional study was designed and data was gathered based on 106 cattle owners and cattle herds; subjected to an interviewer-administered questionnaire and comparative intradermal tuberculin test using a cut-off for positivity of 4 mm, respectively.

**Results:**

Reported levels of cattle and wildlife contact by respondents was at 40%, 58.2% and 1.8%, were relatively proportional to herd level prevalence of cattle BTB at 64.8%, 58.1% and 5.9% in Blue lagoon, Lochinvar and Kazungula respectively. Although 42/106 (39.6%) of cattle owners had heard of BTB, only 3 (7%) had an idea on how the disease was spread. Cattle contact with wildlife was associated with high levels of awareness by cattle owners (χ^2 ^= 43.5, df = 2, *P *< 0.001). Awareness of BTB in low prevalence settings was lower compared to high prevalence settings.

**Conclusions:**

Our study has revealed low levels of awareness among cattle owners on BTB. These results could be useful for policy makers when planning mitigation measures to consider awareness levels by cattle owners for effective implementation. Such information is useful for determining sensitisation programs for cattle owners before mitigation. These results further provide useful insights that disease control is a multi-factorial process with cattle owners as an integral part that can support policy implementation.

## Background

In Zambia, bovine tuberculosis (BTB), caused by *Mycobacterium bovis*, a member of the *Mycobacterium tuberculosis *complex (MTC), has previously been reported in the wildlife-livestock interface areas of the Kafue basin [1-3]. The Kafue basin has a long history as an area with a high prevalence of BTB in Zambian cattle with the wildlife-livestock interface being suggested as the high risk area and the Kafue lechwe antelopes (*Kobus leche Kafuensis*), being the wildlife reservoir hosts [1-5]. The basin is one of the few lucurstrine wetlands supporting close to 300,000 cattle [6] at a carrying density of 50 animals per square kilometre on a 6,000 square kilometre flood plain with a variety of wildlife species whilst the Kafue lechwe antelope form the mega fauna with an estimated population of 44,000 [7].

In wildlife-livestock interface areas, one important control measure to prevent the spread of diseases from known wildlife reservoirs is to restrict wildlife-livestock contacts [8-10]. However, control measures largely depend on the knowledge base of cattle owners for success or failure. Cattle owners play a critical role in the implementation and success of disease control programmes [11]. To our knowledge, no studies on cattle owners' awareness of BTB and other zoonotic diseases have been conducted in Zambia despite livestock production and agriculture in general being the mainstay of the economy after mining and tourism. The basin has been identified as an important area with a high potential for beef production, but this status is seriously threatened by the sustained reports of serious diseases such as BTB [4,12-15].

The impact of tuberculosis (TB) on human health has been devastating worldwide with more than 3.5 million people dying annually from TB with BTB being responsible for 3% of these cases [16]. However, in Zambia, the extent of *M. bovis *involvement in the national tuberculosis burden is unknown. The situation is further compounded by poor or non existent institutional support systems and lack of control and research facilities. The responsibility of controlling infections that are not considered as "diseases of national economic importance" (DNEIs), despite their serious public health effects, lies entirely with cattle owners. It becomes important for cattle owners to acquire a degree of awareness of circulating livestock diseases in their areas notwithstanding the risks they pose, and possible transmission routes to humans, if they are to make informed decisions on diseases suspected to have zoonotic potential.

Risk factors associated with BTB in the Kafue basin have been identified using epidemiological models [1,12]. However, the level of awareness of BTB by local communities is unknown. Studies in industrialized countries have shown that mitigation of BTB in cattle populations can drastically reduce or eradicate the disease in human communities [16-18]. There are indications that mitigation of wildlife-livestock interaction reduces the levels of infection when cattle owners play a central role in planning control measures [10].

The overall objective of this study was to assess and determine cattle owners' awareness of BTB in high and low prevalence areas of the wildlife-livestock interface areas in Zambia.

## Results

### Household characteristics of cattle owners

One hundred and six randomly selected villages that formed the primary sampling units of 106 households and corresponding cattle herds from 3 main study regions of Kazungula (*n *= 23), Lochinvar (*n *= 35) and Blue lagoon (*n *= 48) were selected. A total of 1,487 family members constituted the 106 households from which the minimum number of individuals per household was 2 and the maximum were 39 with an average household having 14 individuals. Cattle owners who entirely depended on their animals as the principal source of their livelihood were 96.2% (102/106), while 3.8% (4/106), were cattle owners who also had extra sources of livelihood. Of the 3.8%, none were in Lochinvar with 2 from Blue lagoon being cotton farmers, while the other 2 from Kazungula were fishermen from Lower Ngwezi, apart from being cattle owners. Ninety eight percent of the households were male headed across the three study areas with only 2 percent being female headed households.

### Awareness and knowledge of tuberculosis by cattle owners

A general overview of results is provided in table 1. Among the cattle owners that were interviewed, (*n *= 64), 60.4% had not heard of bovine tuberculosis, or tuberculosis in animals. Of the cattle owners who had heard of tuberculosis in animals, only 7% (3/64) had an idea on how the disease is spread with 92.9% (39/64) having no basic knowledge of its spread. Among the 3 cattle owners that were aware of the disease, all came from Lochinvar and none from Blue lagoon and Kazungula. Further, 84.9% of the cattle owners were not aware of tuberculosis in wildlife. Among those who were aware of tuberculosis in wildlife (*n *= 16), 15.1%, Lochinvar had a greater majority at 75% (12/16) with the remaining 25% (4/16) in Blue lagoon and none from Kazungula. Cattle owners are particular about who takes care of their animals with preference of taking care and herding cattle being left within close family members by the majority of cattle owners (*n *= 84), 79.3% (Table 1). Awareness of tuberculosis was associated with the experience of having an animal condemned at the abattoir (χ^2 ^= 3.9, df = 1, *P *< 0.05). A significant association was seen when cross tabulating tuberculosis awareness and having a positive herd (χ^2 ^= 7.3, df = 1, *P *< 0.001), indicating that higher awareness is associated with positive herds.

**Table 1:** Cattle owners' response to questionnaire on knowledge of BTB in relation to the wildlife-livestock interface areas (2003/4).

Question	Response	Responders	
		n	%
Have you heard about bovine tuberculosis (BTB)	Yes	42	39.6
	No	64	60.4
			
If YES, do you know how its spread	Yes	3	7.1
	No	39	92.9
			
Are you aware of BTB in wildlife	Yes	16	15.1
	No	90	84.9
			
Type of grazing system practised	Village Resident Herds (VRH)	7	6.6
	Transhumance System (TH)	97	91.5
	Interface System (IFH)	2	1.9
			
Have your cattle been in contact with wildlife	Yes	55	51.9
	No	51	48.1
			
Have you seen your cattle share watering points with wild animals simultaneously	Yes	54	50.9
	No	52	49.1
			
Have you sold an animal in the previous 12 months	Yes	73	68.87
	No	33	31.13
			
Where did the buyers come from	Local buyers from town	18	16.9
	Within the province	17	16.1
	Drove animals "on hoof" to Lusaka	39	36.8
	Can't recall	32	30.2
			
Have you ever had an animal's lungs condemned at an abattoir due to nodular growths and told its TB	Yes	36	33.9
	No	70	66.1

### Epidemiological Parameters

Across the three study areas, transhumance grazing system was the common system practised (Table 2). Herd size was related to the type of grazing system (Table 2). Herd level BTB prevalence in transhumant herd (TH) was comparably higher than the village resident herds (VRH) (Table 2). Only 2 herd owners practised Interface herd grazing system (IFH). There was a significant difference in herd level prevalence of BTB in relation to contact with wildlife based on the area of study (χ^2 ^= 43.5, df = 2, *P *< 0.0001). In Kazungula region, only one cattle owner confirmed having seen his animals come in contact with wild animal species (Table 3). The response by cattle owners and their proportions of affirmatives corresponded with the level of BTB in livestock (Table 3). The effect of proximity to wild animals was further assessed by the sharing of watering points of cattle and wild animals (Table 1) and further by contact (Table 3). Sharing of water between wildlife and cattle was identified as a significant factor for BTB positivity (χ^2 ^= 37.3, df = 2, *P *< 0.0001). In Lochinvar, close to 88% of the animals were reported to have had shared water with wildlife. In Kazungula the animals that were reported to have had shared water with wildlife accounted only for 8% of the studied population.

**Table 2:** Epidemiological characteristics of BTB related to prevalence at herd level across study areas (*n *= 106: August 2003 to February 2004)

Variable	Study area	Median herd size (quartile range)	Herds with BTB prevalence (95% Confidence Interval)
**Grazing strategy**	Village (VRH) (*n *= 7)	42 (39,106)	38.7% (0,84.9)
	
	Transhumant (TH) (*n *= 97)	51 (35,89)	51.6% (39.2,64)
	
	Interface (IFH) (*n *= 2)	61 (26,95)	-

**Overall** **Prevalence**	Across study areas (*n *= 106)	51 (35,89)	49.8% (37.9,61.7)

**Table 3:** Relationship by area of study, of awareness of BTB; contact with wildlife and Herd level prevalence (*n *= 106)

Area of Study	BTB awareness and knowledge		Contact with wildlife		Herd level BTB prevalence at (95% CI)
		
	No. of affirmative response	Proportion of positive response	No. of affirmative response	Proportion of positive response	
Blue Lagoon	19	39.6%	22	40%	64.8% (45.3-84.3)

Lochinvar	15	42.9%	32	58.2%	58.1% (35.2-80.5)

Kazungula	8	8.3%	1*	1.8%	5.9% (0-16.5)

Overall	42	39.7%	55	51.9%	49.8% (37.9-61.7)

*The type of wildlife contact reported in Kazungula was with duikers, rabbits and not lechwe antelopes.

## Discussion

Our results indicate that 39.6% of cattle owners were cognisant of bovine tuberculosis across the study areas. However, only 7% had basic knowledge of the disease in terms of its mode of spread. Further, all those who knew how the disease was spread were cattle owners based in Lochinvar which is a high prevalence setting [3,12,14] and none from Kazungula (a low prevalence setting) [1]. Blue lagoon despite being in a high prevalence setting reported no cattle owner with basic knowledge on how tuberculosis is spread. Considering that both Lochinvar and Blue lagoon are in the high prevalence setting [1], these findings intimate area variations as a platform evincing different factors of BTB awareness, albeit the similarity in prevalence setting. This variation in the levels of awareness between two regions sharing high prevalence and similar ecological settings may suggest the presence of different underlying factors unique to the two areas. Notable about Lochinvar is the presence of a defunct abattoir [19] which was operational between 1968 to 1972 for specifically screening wild animals for tuberculosis and other infections [13,14,20]. This was a point of reference by Lochinvar cattle owners who had better knowledge of the disease than those in other areas. These results suggest that to a larger extent, area deterministic factors may have additional effects on disease awareness levels by cattle owners. Further, history of wildlife culling in the 1970s, to detect BTB in lechwe antelopes [13,14,20] in Lochinvar may have created an extra source of information to the local cattle owners in this area.

Based on earlier epidemiological studies, high prevalence of BTB appear to have had an effect on the awareness of the disease [21]. Other studies have indicated that the level of disease awareness among famers is related to the prevalence of the disease [10]. However, these observations are related to area dependant factors that influence the existence of high prevalence, i.e., the presence of wildlife reservoirs of the disease [10,22]. Such underlying factors may be sufficient determinants in closely related ecological areas like in Lochinvar and Blue lagoon, with both areas sustaining high prevalence settings, hence having a much higher level of cattle owners' awareness of BTB than Kazungula with a different ecological setting.

Despite the lack of awareness on BTB by most cattle owners, they were worried about introducing diseases into their cattle herds, as over one third of the owners had experienced the pain of taking an animal to the abattoir and to have its plucks condemned (Table 1), and sometimes whole carcasses condemnations. This was of particular concern to cattle owners as it resulted in direct loss of income, and these formed the core majority of the cattle owners who were aware of tuberculosis in both the high and low prevalence settings. Further, the study found a strong association between having a BTB positive herd on skin test and level of awareness by the cattle owners (χ^2 ^= 7.3, df = 1, *P *< 0.001).

As herd size increased, cattle owners tend to take their animals into the plains joining into the practice of transhumance grazing which brings their animals in contact with wildlife [23].

During such periods, livestock and wild animals share drinking points. Sharing of water between wildlife and cattle was identified as a significant factor for BTB positivity. However, this may have been a bit subjective considering that not every cattle owner may have had seen their cattle sharing water points with wild animals simultaneously. However, during the questionnaire interview, the family members sat as a group to give as much accurate information as possible and the herd boys were available in most cases and further the family members accounted for more than 80% of the people who herded the animals consolidating the accuracy of the information.

Studies elsewhere have shown that closeness to disease increased concern among cattle owners [10]. However, this was not in agreement with what is obtaining in the high BTB prevalence area of Blue lagoon, where despite high prevalence; the interest shown was low, but similar findings are congruent with what is obtaining in Lochinvar area [10].

Our results are important in managing not only BTB in complex pastoral communities where perceptions to disease occurrence vary and where standard disease control measures may fail to achieve desired results. However, our results intimate that disease control in livestock should incorporate socio aspects. Our findings, where cattle owners with good knowledge of the disease were those with prior exposure to BTB control activities merits further exploitation of farmer supported programs and actions in areas where such knowledge is deficient. Further from this study, the major factors that were identified to be influencing knowledge gaps between different BTB prevalence settings were not only plausible biologically, but also socially. This underscores the importance of disease awareness campaigns. This should take form in farmer education, farmer supported actions and participation in disease extension services. Such active participation in disease control activities will develop the farmers' interests further assisting disease control experts when adopting workable methodologies aimed at controlling livestock diseases such as BTB in diverse farming communities with varying levels of disease perceptions among cattle owners. In summation, these are key lessons that may be relevant for other settings where a similar situation may exist before standard disease control measures through a multifaceted approach involving Veterinarians and Sociologists are envisaged.

The validity of the data may be affected by interviewer bias, but this was avoided by limiting only to two persons as interviewers during the whole period of the study. In order to improve the accuracy of the data collected during these interviews, the data relevant for the TB survey were collected simultaneously with data collected for other TB and *Brucella *questionnaires[12,24,25] Further, the questionnaires were pretested to avoid confounding questions and to test for clarity of the questions among other aspects. Our study was designed and conducted as cross sectional in nature. However, this design has limitations of considering events at a particular point in time. Perceptions differ with time and the lack of information before the abattoir was built in Lochinvar denied the study comparative reference. However, the findings represent prevailing levels of awareness by cattle owners in high and low prevalence settings in relation to epidemiological characteristics of BTB at the time of the study. Additionally, we tried to reduce recall bias by basing questions to the preceding 12 months before the study period. In case this study was to be conducted again, the questionnaire design would include both dichotomous variables from closed questions and open questions especially were the range of responses is not known.

All in all, our results indicate a relatively good level of disease awareness to those cattle owners in areas of high prevalence settings, peculiarly in areas augmented by existing secondary factors, activities and epidemiological characteristics related to the disease under consideration. These findings further highlight the need to sensitize cattle owners on prevailing diseases, drawing on their support, both as counterpart contact personnel for extension services as well as supporters of the disease control programs.

## Conclusions

Overall, our study has revealed low levels of awareness among cattle owners on BTB. These results could be useful for policy makers involved in planning mitigation measures to consider awareness levels by cattle owners for effective implementation. Such information is useful for determining sensitisation programs for cattle owners before mitigation. These results further provide useful insights that disease control is a multi-factorial process with cattle owners as an integral part that can support policy implementation. Based on these results we recommend that all future livestock disease control strategies should be farmer based, or should provide an element of determining the level of knowledge of the disease by cattle owners since most of the diseases are associated with cattle husbandry systems.

## Methods

### Selection of Study Areas

**Figure 1:**
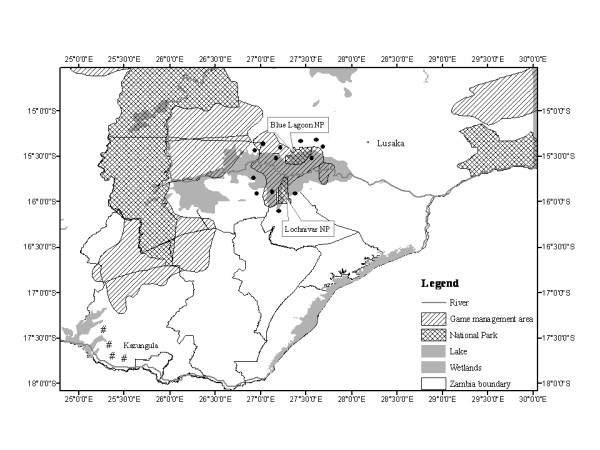
**Location of study sites; High prevalence setting were around Blue Lagoon and Lochnivar NPs (black circle), Low prevalence setting were in Kazungula (#)**.

Three pastoral areas were selected; two from the Kafue basin (high prevalence setting) in the wildlife-livestock interface areas and one area outside the wildlife-livestock interface in Kazungula district (low prevalence setting) (Figure 1). The Kafue basin is a floodplain of about 6,000 km^2 ^[26-28] comprising Lochinvar (410 km^2^), Blue Lagoon National Park (420 km^2^) and the Game Management Areas (GMAs) (5,175 km^2^) [29]. The interface areas of the Kafue basin National Parks are endowed with wildlife, particularly the Kafue lechwe antelope (*Kobus leche Kafuensis*) which interacts freely and easily with livestock (cattle). Kazungula District was added for comparative purposes based on similar cattle rearing practices although the reported levels of wildlife interaction with cattle in this area are very minimal. However, both communities practice transhumance grazing strategies (a grazing system where animals are taken to the wetlands during the dry season in search of grass, and taken back to the uplands when floods occur during the rain season). Kazungula district is located 400 km south of the Kafue basin and lies along the Zambezi River basin (Figure 1).

### Designing the Study

The study was conducted as a cross-sectional study from August 2003 to February 2004. Currently, another bigger study along the same lines is being conducted. The information in this particular paper is being provided to give a comparative reference point to the new data which shall come out from the new study. Due to lack of comprehensive information on the number of cattle owners and corresponding cattle herds in the study areas, we had to first conduct a baseline study. During baseline studies, we discovered that cattle ownership in the intended study areas was a complex issue. In order to increase the independency of ownership of the cattle herds, all factors related to herd ownership had to be considered in the definition of a herd. A herd was the study unit of interest and in certain cases, a 'herd' consisted of village clusters or grazing groups, after taking into account the issue of ownership. Where more than one person owned cattle within that herd, ownership was allotted to only one person (for study purposes, as the owner) as these animals were exposed to similar factors. In villages where animals grazed closely despite belonging to different owners, only one cattle owner was randomly chosen in that village. In certain villages, it was found that one person can have his cattle in different herds. In other villages, they had "super herds" (multi owned herds), where all the individuals in that village shared the responsibilities of keeping the animals, and in such situations, only one "super herd" existed in that village and for study purposes it was considered as a single herd and such herds were allotted to only one owner for study purposes. Based on the baseline study, and after taking all factors into consideration, we estimated that there were approximately 110 cattle herds in the Blue Lagoon area, 100 in Lochinvar and 50 in Kazungula. During the baseline study, all cattle owners in the targeted study areas were listed as the targeted population. This population of cattle owners and cattle herds constituted the study population from which actual sampling was conducted (sample population).

Assuming low heterogeneity between herds, we used a detection power (1-β) of 90%, the level of significance (α) at 95% and the desired absolute precision at 5%. We further assumed the sensitivity and specificity of the comparative intradermal tuberculin test (CITT) to be 80% and 100%, respectively [30,31]. The BTB prevalence previously reported for cattle in Zambia varies from 10% to 20% at animal level [2,21]. We therefore assumed an average of 15% as BTB animal prevalence with herd level prevalence being estimated at 30%. The average herd size was assumed to be at 100 animals. We thus planned to sample individual cattle owners (for questionnaire administration) and cattle herds (for CITT) from a sampling fraction of 10%. Based on these assumptions, we used *Herdacc*™ Version 3 [32] to estimate herd specificity (HSp) and herd sensitivity (HSe). Our predicted HSp and HSe were 100% and 73.9% at 10% sampling fraction, where a herd was classified positive if at least one animal tested positive on CITT. Thus applying the estimates in the sample size calculation formula for simple random sampling, and correcting for a finite population we planned to sample 125 herds represented as 53, 48 and 24 herds for Blue Lagoon, Lochinvar and Kazungula, respectively. It was not possible to sample 125 herds in each study site given the complexity of an independent herd that was considered as an independent epidemiological unit. Further, some areas had few cattle herds than other areas. To select this number of cattle owners and cattle herds and to avoid selection bias, a simple random mechanism of choosing herds was designed using a lottery system. In each study area, cattle herds (also representing owners) were given numbers on a piece of paper. These numbers were then put in a suitable receptacle from which random selection of herds was done, without replacement. In areas where farmers were un-cooperative, other herds having similar exposure factors, such as sharing grazing land and water and having similar management strategies, were chosen as replacement herds.

### Data collection: conducting a structured questionnaire Survey

Data was collected using "closed-ended", pre-tested questionnaires (tested during baseline studies) written both in English and the local language used in the study area (see Additional file 1). The questionnaires were administered by "face to face" interviews mainly by the principal researcher who is a native speaker of the local language spoken in the study areas. The interviews took between 20 to 30 minutes and were done at the respondent's convenience in connection to the tuberculinisation exercises. The questionnaire was divided into two main sections. The first section involved surveying animal tuberculosis and this included gathering cattle inventory and demographical data parameters, cattle grazing systems, movement patterns, wildlife contact, animal production, marketing systems and knowledge of animal diseases. The second section detailed the cattle owners' descriptions and knowledge of cattle tuberculosis experienced in their herds as well as knowledge of the disease in humans. In both sections, questions were asked for a period preceding the last twelve months to avoid poorly recalled data. Scientific and ethical clearance to conduct this study was obtained from the University of Zambia (UNZA), Research Ethics Committee with Assurance NO. FWA00000338 IRB00001131 of IOR G0000774 (Ref: 007-02-04).

### Biological data collection in cattle; tuberculin skin test

In order to determine the prevalence of BTB in cattle, the comparative intradermal tuberculin (CITT) test was applied. The procedure was conducted as described in the OIE manual [33]. Two circular areas of about 2 cm^2 ^diameter, about 12 to 15 cm apart, on the cervical area of the skin, were clipped, washed with soap and disinfected with 70% ethanol. The initial skin thickness was measured followed by a subcutaneous injection of 0.1 ml of 5000 IU bovine and avian purified protein derivatives (PPD) manufactured by ID Lelystad the Netherlands. The results of hyper-sensitisation were read after 72 hours by again measuring the skin thickness. A strict standard level of interpretation was used to classify reactors according to the OIE manual [33]. Negative reactors were indicated by increases in differential skin thickness increment of less than 2 mm when the avian reading was subtracted from the bovine reading. Inconclusive reactors were indicated by differential skin thickness increment of between 2 mm and 4 mm, while a positive reaction was indicated by differential skin increment of more than 4 mm. Further still, a negative reactor was identified when there was no reaction to bovine tuberculin, or a positive or inconclusive reaction to bovine tuberculin that was equal to, or less than a positive or inconclusive reaction in avian test and also when negative to both [33]. A herd was classified positive if at least one animal in the herd tested positive on CITT.

### Statistical analyses

The database was established in Excel ^® ^before transferring to Stata SE/10 for Windows (Stata Corp. College Station, TX, USA). All data handling, validating, cleaning and coding was done in excel spread sheets and all analysis were conducted using the Stata SE/10 for Windows (Stata Corp. College Station, TX, USA). Herd level data included information about herd structure, wildlife contact, ecological and management factors with possible influence on BTB. This information was compared to what the cattle owners perceived and what information they had on tuberculosis factors especially in relation to the wildlife-livestock interaction. Herd level prevalence estimates for BTB with confidence intervals were computed using the survey command estimates in Stata with adjustments for strata (study area) as described by Dohoo and coworkers [34]. Socio-demographical variables describing respondents in the region were summarized to characterize cattle owners. In order to consider cattle owners' concerns regarding disease within the context of their areas, factor analysis was used. The closed ended questionnaire allowed the use of quantitative data through the coding of the relevant descriptors under study.

This paper conforms with the reporting standards outlined in the STROBE (Strengthening the Reporting of Observational studies in Epidemiology) statement [35].

## Abbreviations

BTB: bovine tuberculosis; CITT: comapartive intradermal tuberculin test; DNEIs: diseases of national economic importance; GMAs: Game Management Areas; HSe: herd sensitivity; HSp: herd specificity; IFH: Interface herds; MTC: *Mycobacterium tuberculosis *complex; OIE: Office international des epizooties; TH: Transhumance herds; VRH: Village resident herds.

## Authors' contributions

MM contributed to the design, data collection, tuberculinisation of cattle, analysis of data and drafting of the manuscript. JBM contributed to the design, supervision of field work, drafting and writing of manuscript. HMM contributed to field studies during tuberculinisation, drafting and reviewing of the manuscript. CK contributed to drafting of the manuscript. ES contributed to conception and design, data analysis and the writing of manuscript. MT contributed to supervision of the project, acquisition of parts of the funds and writing of the manuscript and important intellectual contribution. All authors have read and approved the final manuscript.

## Supplementary Material

Additional file 1 **A cross-sectional survey on human and animal tuberculosis to determine the risk factors and disease awareness by cattle owners**. The file contains a questionnaire form that was used to assess the awareness of tuberculosis by farmers/cattle owners in their area among other epidemiological factors and data that was collected. Click here for file
