# Supplementary material for: Cattle owners' awareness of bovine tuberculosis in high and low prevalence settings of the wildlife-livestock interface areas in Zambia
Source: BMC Vet Res. 2010 Apr 20;6:21. doi: 10.1186/1746-6148-6-21 (PMC2874791; doi:10.1186/1746-6148-6-21)
Supplement: Additional file 1 — A cross-sectional survey on human and animal tuberculosis to determine the risk factors and disease awareness by cattle owners. The file contains a questionnaire form that was used to assess the awareness of tuberculosis by farmers/cattle owners in their area among other epidemiological factors and data that was collected. [file 1746-6148-6-21-S1.DOC]

**A CROSS-SECTIONAL SURVEY ON HUMAN AND ANIMAL TUBERCULOSIS TO DETERMINE THE RISK FACTORS AND DISEASE AWARENESS BY CATTLE OWNERS**

***Department of Disease Control, School of veterinary Medicine, University of Zambia, P.O Box 32379, Lusaka Zambia, Tele-fax 292737***

**SURVEY ON ANIMAL TUBERCULOSIS**

*SECTION ONE:* ***IDENTIFICATION***

Date:……………………………………………………………………………………

Village / Farm Sampling No:…………………………………………………………...

Area / Location:…………………………………………………………………………

Owner’s name:…………………………………………………………………………

Address:………………………………………………………………………………..

Name of the person interviewed:……………………………………………………...

Relation of the person interviewed:

1. Owner 2.Family member 3. Caretaker

Name of interviewer:…………………………………………………………………….

Q 1.

Breakdown of Herd structure:

| Cattle and age category | Number |
| --- | --- |
| Female calves < (less than) 1 year |  |
| Male calves < (less than) 1 year |  |
| Male cattle between 2-3 years |  |
| Female cattle between 2-3 years |  |
| Female cattle over 4 years |  |
| Male cattle over 4 years |  |

Q 2.

Who is primarily responsible for looking after the animals?

1. Owner / family member………………………………………………………….
2. Hired caretaker………………………………………………………………….
3. Both 0 and 1……………………………………………………………………….
4. Others specify……………………………………………………………………

Q 3.

Do you receive any Veterinary services?

1. No…………………………………………………………………………….
2. Yes……………………………………………………………………………..

*SECTION TWO:* ***CATTLE FEEDING PATTERNS, WILDLIFE CONTACT & MOVEMENTS***

Q 4.

What type of feeding / grazing system do you practice?

1. Grazing from own fields / paddocks…………………………………………..
2. Grazing from communal pastures……………………………………………..
3. Communal and own pasture grazing…………………………………………..
4. Other Describe……………………….…………………………………………..

Q 5.

Do you move your animals to the plains on certain times of the year for search of grazing land?

1. No………………………………………………………………………….
2. Yes………………………………………………………………………….

Q 6.

If yes, do they come in contact with wild animals in the plains?

1. No………………………………………………………………………….
2. Yes………………………………………………………………………….

Q 7.

Where do your animals drink water?

1. Own watering points…………………………………………………………….
2. Shared / Communal watering points…………………………………………….
3. Own and communal watering points……………………………………………

Q 8.

Do your animals share drinking water with wild animals simulatenously?

1. No……………………………………………………………………………..
2. Yes……………………………………………………………………………

Q 9.

Have you sold any animals in the last twelve months?

1. No…………………………………………………………………………………
2. Yes……………………………………………………………………………….

Q 10.

If yes, where did you sell your animals, or where did the buyers come from?

1. Within the neighbouring villages………………………………………………..
2. Within the town…………………………………………………………………..
3. Within the province………………………………………………………………
4. Outside the province……………………………………………………………..
5. Others Specify……………………………………………………………………

Q 11.

Do you sell your milk?

1. Within the neighbourhood……………………………………………………….
2. Within the town…………………………………………………………………..
3. Within the province……………………………………………………………….
4. Outside the province………………………………………………………………
5. Others specify……………………………………………………………………..

*SECTION THREE:* ***KNOWLEDGE OF THE DISEASE***

Q 12.

Did any animal die in your herd in the last 12 months?

1. No……………………………………………………………………………
2. Yes……………………………………………………………………………

Q 13.

Were there any coughing animals in your herd in the last 12 months?

1. No…………………………………………………………………………..
2. Yes…………………………………………………………………………..

Q 14.

Do you have very thin and emaciated (wasted away) animals in your herd?

1. No…………………………………………………………………………...
2. Yes……………………………………………………………………………

Q 15.

Did any animal showing the signs in Q13 & 14 above die?

1. No………………………………………………………………………………
2. Yes……………………………………………………………………………..

Q 16.

Have you ever herd about bovine tuberculosis (BTB)?

1. No……………………………………………………………………………
2. Yes……………………………………………………………………………

Q 17.

If YES, do you know how its spread?

1. No……………………………………………………………………………
2. Yes……………………………………………………………………………

Q 18

Are you aware of BTB in wildlife?

1. No……………………………………………………………………………
2. Yes……………………………………………………………………………

Q19

Have you ever thrown away lungs or part of lungs due to nodular (hard growths) on them or at an Abattoir has any of your animal(s) been condemned for suspected TB?

1. No………………………………………………………………………
2. Yes………………………………………………………………………

**SURVEY ON HUMAN TUBERCULOSIS**

*SECTION ONE:* ***IDENTIFICATION***

Date:………………………………………….

Area;………………………………………….Sampling No:……………………………

Name:……………………………… ……………………….Sex…………..Age………..

Number in the household:…………………………………………………………………

Q 1.

Occupation

1. Farmer…………………………………………………………………
2. Shepherd………………………………………………………………
3. Others Specify…………………………………………………………

*SECTION TWO****: OWNERSHIP & CONTACT WITH ANIMALS AND THEIR PRODUCTS***

Q 2.

Do you keep cattle?

1. No…………………………………………………………………………….
2. Yes………………………………………………………………………………

Q 3.

Have you been in contact with animals recently?

1. No……………………………………………………………………….
2. Yes……………………………………………………………………….

Q 4.

If yes, what type of contact was it?

1. Herding the animals………………………………………………….
2. Milking the animals………………………………………………..
3. Others specify…………………………………………………………..

Q 5.

Do you handle animal products?

1. No……………………………………………………………….
2. Yes…………………………………………………………………..
3. If yes, specify…………………………………………………….

Q 6.

Do you drink fresh milk?

1. No………………………………………………………………..
2. Yes………………………………………………………………..

Q 7.

If you do, how often do you drink fresh milk?

1. At least every day……………………………………………………
2. At least every week………………………………………………….
3. At least every month…………………………………………………
4. Others specify………………………………………………………..

Q 8.

Do you boil your milk before drinking?

1. No………………………………………………………………….
2. Yes………………………………………………………………..

Q 9.

Do you process sour milk?

1. No………………………………………………………………………
2. Yes……………………………………………………………………..

Q 10.

If yes, do you process sour milk from boiled milk?

1. No……………………………………………………………………………
2. Yes………………………………………………………………………….

Q 11.

Do you drink sour milk?

1. At least every day………………………………………………………
2. At least every week…………………………………………………..
3. At least every month………………………………………………………
4. Never………………………………………………………………….
5. Other specify…………………………………………………………

Q 12.

What is your main source of milk?

1. Own animals…………………………………………………………….
2. Others, i.e. neighbors…………………………………………………….
3. Others specify…………………………………………………………….

*SECTION THREE:* ***KNOWLEDGE OF THE DISEASE***

Q 13.

Have you ever heard of a disease called tuberculosis (TB)?

1. No………………………………………………………………………
2. Yes……………………………………………………………………..

Q. 14.

Has any member of your family received treatment for tuberculosis (TB) in the last 12 months?

1. No……………………………………………………………………………
2. Yes…………………………………………………………………………..

Q 15.

If yes, at what approximate **age** did they receive the TB treatment?

Specify…………………………………..……………………………………

Q16.

Are all members of your household vaccinated with BCG?

1. No……………………………………………………………..
2. Yes…………………………………………………………….
3. Don’t know……………………………………………………

**Additional Information**

**HERD TB STATUS:………………………………………………………**
